# The Influence of the Morphology and Mechanical Properties of Polymer Inclusion Membranes (PIMs) on Zinc Ion Separation from Aqueous Solutions

**DOI:** 10.3390/polym10020134

**Published:** 2018-01-30

**Authors:** Katarzyna Witt, Elzbieta Radzyminska-Lenarcik, Artur Kosciuszko, Magdalena Gierszewska, Kamil Ziuziakowski

**Affiliations:** 1 Faculty of Chemical Technology and Engineering, UTP University of Sciences and Technology, PL 85326 Bydgoszcz, Poland; 2 Faculty of Mechanical Engineering, UTP University of Sciences and Technology, PL 85796 Bydgoszcz, Poland; Artur.Kosciuszko@utp.edu.pl; 3 Faculty of Chemistry, Nicolaus Copernicus University in Torun, PL 87100 Torun, Poland; mgd@chem.umk.pl; 4 Faculty of Chemistry, Adam Mickiewicz University in Poznan, PL 61614 Poznan, Poland; ziuziakowski@gmail.com; 5 Przedsiebiorstwo Wielobranzowe GALKOR Sp. z o.o., PL 86010 Koronowo, Poland

**Keywords:** polymer inclusion membrane, metal ion separation, zinc(II), membrane mechanical properties, membrane morphology

## Abstract

The transport of Zn(II) ions across polymer inclusion membranes (PIMs) with acetylacetone (ACAC) or di(2-ethylhexyl)phosphoric acid (D_2_EHPA) as carriers was studied. Polymeric membranes consisting of polyvinylchloride (PVC) as the support, *bis*(2-ethylhexyl)adipate (DAO) as plasticizer, and ACAC or D_2_EHPA as ion carriers were investigated. The highest recovery factors for Zn(II) ions were observed in the case of a membrane containing 20% acac (99.6%) and 60% D_2_EHPA (56.3%). The prepared PIMs were examined using atomic force microscopy (AFM) techniques. Their mechanical properties were also determined. The influence of membrane morphology and mechanical properties on the zinc transport process was discussed.

## 1. Introduction

Polymer inclusion membranes (PIMs) were first prepared as Solvent Polymeric Membranes (SPMs), in 1963, by Bloch [1]. They were obtained by pouring out a polymer-carrier solution on paper. Although the enhancement of the membrane with paper improved its mechanical properties, it also hindered the diffusion of substances across the membrane. Twenty years later, Sugiura [2] improved this technique by introducing an additional component (plasticizer). The added component appeared to improve the polymer film in terms of mechanical strength, so paper was no longer required. To obtain a PIM, a clear quaternary solution (polymer, solvent, plasticizer, and carrier) is poured out on a glass plate and the solvent is allowed to evaporate. Then the membrane is placed in distilled water [3,4,5]. The obtained membranes are characterized by a negligible loss of carrier during pertraction. Moreover, in this procedure only low amounts of potentially hazardous chemicals are used. The chemical composition and membrane thickness can be easily modified [6]. PIMs are also characterized by higher mechanical strength and stability in comparison with other liquid membranes (SLMs) [7].

The role of the plasticizer is to improve the PIM in terms of elasticity and mechanical strength, as it penetrates into the polymer molecules and mitigates the intermolecular forces. This results in greater distances between the polymer molecules. Although a considerable number of plasticizers are available on the market, only a few of them were examined with regard to application in PIMs. The most frequently used plasticizers are: *o*-nitrophenyloctyl ether, *o*-nitrophenylpentyl ether, dioctyl adipate, dioctyl phthalate, and ethyltrialkylammonium chloride (Aliquat 336), which can act as a plasticizer and an anion carrier [8,9,10]. Polymer matrices made of polyvinyl chloride (PVC), polyvinylidenefluoride (PVDF), cellulose triacetate (CTA), or cellulose tributyrate (CTB) are used in the majority of studies devoted to PIMs [8,11,12]. Metal ion carriers in transport across PIMs are chemical compounds which are easily soluble in a liquid membrane, insoluble in aqueous solutions and which selectively and reversibly interact with the transported component [13]. Generally, the carriers are the same organic ligands as the extractants in solvent extraction [12,14,15,16,17,18,19].

The rapid development of industry, including its nuclear and hydrometallurgical branches, resulted in intensified studies on PIMs intended to be utilized in the processes of recovery, separation and concentration of metal ion from post-production waste solutions. The membranes are effectively used for the recovery of s-, p-, d-, and f-electron metal ions, but the PIMs which enable the recovery and separation of heavy metals enjoy the greatest interest [8,9,20]. The PIMs are also used for a more thorough purification of solutions formed in heavy-metal waste treatment by means of conventional methods (precipitation with slaked lime, ion exchange) [21].

Zinc and zinc compounds are used in various branches of industry. Metallic zinc is used for the preparation of anti-corrosive coatings for metal objects, production of alloys (such as brass), so-called dry cells (batteries) and anti-corrosive paints [22]. It takes between 4 and 9 kg of zinc to make a modern car. Zinc compounds are used in the production of medicines and cosmetics. ZnO is a component of varnishes and paints and is also used in production of tires [23,24]. Industrial wastes with a content of zinc, such as ashes, sludges, and liquid wastes, are a separate, very important issue: these are now regarded as metal-bearing materials. The obtaining of zinc from refuse using PIMs and other methods has been reported by a number of authors [25,26,27].

In 2000, the European Union established the Water Framework Directive. Its objective is for the EU member states to achieve good qualitative and quantitative status of water bodies and inland ecosystems which depend on them. The authors of the document had in mind to obtain a good ecological status of waters by various methods, also by reduction or, if possible, elimination of the emissions of highly hazardous substances formed in industrial processes, such as heavy metals, including lead, cadmium, chromium, copper, nickel, or zinc. This legislative measure clearly indicates the necessity of devising procedures resulting in a reduction of environmental emissions of these metals [28].

Given the growing demand for zinc, its high purchase price and the requirement to comply with environmental protection legislation, more than 30% of the metal’s total production volume is obtained by means of various recycling methods [29].

Taking into account the above mentioned facts, there is a clear preference of zinc production methods which are based on the recycling of waste or solutions. Processes where PIMs with metal ion carriers are used are also of interest to researchers. The actual studies are intended to find new, effective metal cation carriers which will be selective and characterized by affinity to specific metals.

High selectivity for zinc is shown by organophosphorus derivatives, such as: di(2-ethylhexyl)phosphoric acid (D_2_EHPA) [30], di(2,2,4-trimethylpentyl)phosphinic acid (Cyanex 272) [31], di(2-ethylhexyl)thiophosphoric acid (DTPA) [32], and di(2,2,4-trimethylphenyl)dithiophosphinic acid (Cyanex 301) [33]. Other potentially good carriers include derivatives of β-diketones, such as 1-phenyl-3-isoheptyl-1,3-propanedione (LIX-54) [34] and 1-phenyldecane-1,3-dione (LIX 54-100) [35]. LIX 54 as carrier is able to separate zinc and nickel from mildly alkaline environments. For example, one scientific report describes the transport of three metals (Cd, Pb, and Zn) across membranes prepared from cellulose triacetate (CTA), 2-nitrophenyloctylether (2-NPOE) with D_2_EHPA or Aliquat 336 as the metal ion carriers. The feed and receiving phases were aqueous solutions of metal ions with 0.1 M NaNO_3_ and 0.1 M HNO_3_ (for D_2_EHPA) or 0.5 M NaCl and 0.1 M HClO_4_ (for Aliquat 336), respectively. Zinc recovery rate from the metal ion mixture with the use of such membranes was 91.02% for the membrane with D_2_EHPA and 56.34% for the membrane with Aliquat 336, respectively [36]. Kolev et al. used a PVC-based membrane with D_2_EHPA as carrier for zinc transport [37]. The feed phase was a solution of 22 mg/L of Zn(II) in 1.0 M acetic acid and acetate buffer was used for maintaining a pH of 3.0. The receiving phase was 0.1 or 1.0 M HCl. 

In addition to known commercial carriers, new selective carriers are gaining increased interest; the materials are tested in various environments. As carriers of CTA-based membranes, Baczynska et al. used three phosphonium ionic liquids (Cyphos IL101, Cyphos IL104, Cyphos IL167) [38]. The authors of the present paper have determined that zinc is effectively transported through the investigated PIMs. Arslana et al. proved that sodium diethyldithiocarbamate (NaDDTC) is an effective, innovative zinc ion carrier, thanks to which transport across the membranes based on CTA and o-NPOE resulted in the 96% recovery of Zn [39]. The process was carried out at a pH 5.04 and the receiving phase was 0.5 M HCl.

In their earlier paper, the authors discussed zinc transport across a CTA-based membrane composed of o-nitrophenyl pentyl ether (*o*-NPPE) as plasticizer doped with 1-alkylimidazoles [40]. The process was effected from a ternary mixture comprising Zn(II)–Co(II)–Ni(II) and from a binary mixture of Zn(II)–Ni(II). Better results of zinc recovery were obtained in the latter case (90%). In their next paper, the authors used carriers in the form of β-diketone derivatives [41]. The compounds proved to be effective in zinc recovery from the quaternary mixture Zn(II)–Cu(II)–Co(II)–Ni(II).

In the present paper, the authors investigated the dependence of the physical and chemical properties of two types of PVC-based membranes on their chemical composition and their efficiency in Zn(II) ion separation in transport across the membranes. The membranes were obtained by solution casting method. Acetylacetone (ACAC) and di(2-ethylhexyl)phosphoric acid (D_2_EHPA) were used as the cation carriers. The membranes were also studied in order to obtain information on their structure. The membrane morphology (pore size and roughness) was investigated by atomic force microscopy (AFM). Such mechanical properties as thickness, tensile strength, and glass transition temperature, were also investigated. An attempt was made to explain the influence of the physical and chemical properties of the membranes on Zn(II) ion transport efficiency.

The structure of the PVC membranes has so far only been studied for commercial carriers (Aliquat 336, TOA, Cyphos^®^ IL 104, D_2_EHPA) [4,37,42].

## 2. Experimental

### 2.1. Reagents

The inorganic chemicals, i.e., ammonia as well as ammonium nitrate, sodium nitrate and nitric acid were of analytical grade and were purchased from Avantor Company (Gliwice, Poland). Zinc(II) nitrate standard solution (1000 mg/L) was purchased from Sigma-Aldrich Company (Poznan, Poland). The aqueous solutions were prepared with a double-distilled water the conductivity of which was 0.1 S/m. 

The organic reagent, i.e., polyvinyl chloride (PVC) suspension, with an average molecular weight of 72,000, was obtained from ANWIL Company, Wloclawek, Poland. Acetylacetone (ACAC) and tetrahydrofurane (both of analytical grade) were purchased from Avantor Company (Gliwice, Poland) and were used without further purification. The di(2-ethylhexyl)phosphoric acid (D_2_EHPA) and *bis*(2-ethylhexyl)adipate (DAO) were purchased from Sigma-Aldrich Company (Poznan, Poland).

### 2.2. Membrane Preparation and Transport Experiments

**Figure 1:**
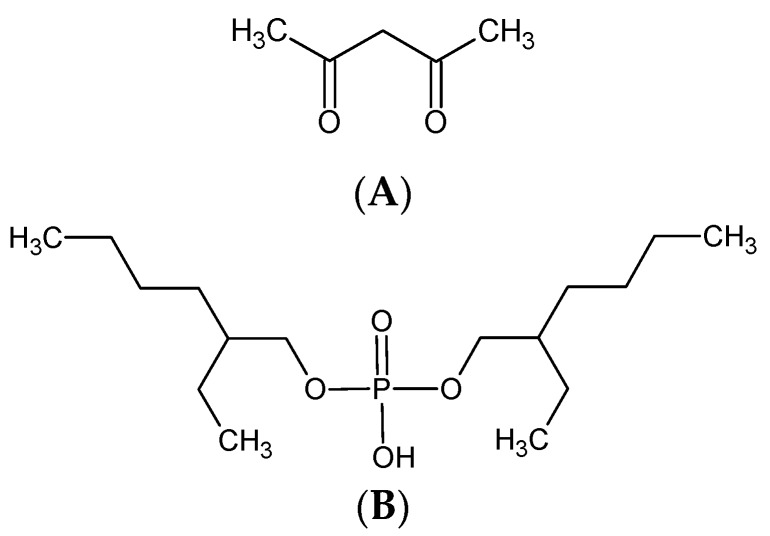
Structure of acetylacetone (**A**) and di(2-ethylhexyl) phosphoric acid (**B**).

At first, a ternary mixture in tetrahydrofurane was prepared using PVC, DAO, and ACAC (Figure 1A) or D_2_EHPA (Figure 1B) as ion carrier.

The obtained mixture was poured out on an ANUMBRA self-levelling dish. After the evaporation of the tetrahydrofurane, 24 h later, the membranes were removed from the glass surface and immersed in distilled water for 12 h to obtain a homogeneous film structure. A total of eleven different membranes was obtained. Their quantitative compositions are presented in Table 1. In order to initiate the process of transport twice in the same conditions, in the case of each membrane, two samples were cut out from the original membrane films.

The transport experiments were carried out in a permeation module cell, which was presented in the authors’ earlier paper [41]. The membrane film (having a surface area of 4.4 cm^2^) was tightly clamped between two cell compartments. Both the feed and the receiving aqueous phases (45 mL each) were mechanically stirred at 600 rpm. Distilled water was used as the receiving phase. The PIMs transport experiments were carried out at 20 ± 0.2 °C. Small samples of the aqueous receiving phase were taken periodically via a sampling port, equipped with a syringe, and analyzed by atomic absorption spectroscopy (AAS Spectrometer, AAS 240FS, Agilent, Santa Clara, CA, USA) to determine zinc(II) concentration. The pH of the feed phase was kept constant using an ammonia buffer and controlled by means of a pH-meter (pH meter, CX-731 Elmetron, Zabrze, Poland), with a combination pH electrode (ERH-126, Hydromet, Bytom, Poland).

### 2.3. AFM Analysis

The synthesized PIMs were examined by an Atomic-force MultiMode Scanning Probe Microscope IIIa (AFM) (Digital Instruments Veeco Metrology Group, Santa Barbara, CA, USA) operating in the tapping mode, in air, at a room temperature. The membranes were visualized in a two-dimensional and in a three-dimensional form, sizes 1.0 × 1.0 μm, 5.0 × 5.0 μm and 10.0 × 10.0 μm.

### 2.4. Tests of Mechanical Properties of PIMs

Mean thickness of all membranes was determined after measuring the test samples with the manual precision thickness gauge Panametrics^®^ Magna-Mike^®^ 8500 (San Diego, CA, USA). The mean value resulted from 10 measurements, made at randomly selected points located on the surface of each membrane.

Mechanical properties of the selected membranes were examined by static extension using the universal testing machine Z030 from Zwick/Roell (Ulm, Germany), equipped with a measurement head, at a nominal force of 500 N. Extension rate during calculation of the modulus of elasticity was 1 mm/min. The extension test was then performed at a rate of 50 mm/min until the breaking of the samples. The test samples were in the shape of a dumbbell, obtained by cut out from the membrane. The total sample length was 82 mm and the length of the part limited by parallel lines was 38 mm. The sample width was 15 mm at the ends and 10 mm in its narrow section, the grips were 50 mm apart initially. The measurements were carried out at 20 °C. Mean values of the mechanical properties were determined based of the tests performed on 5 samples. 

The glass-transition temperature of each membrane samples was measured by scanning differential calorimetry, using the DSC 2014 Polyma apparatus from Netzsch, Selb, Germany. The samples (7–9 mg) were heated under nitrogen atmosphere from room temperature to 120 °C to remove any thermal or mechanical history of the material. Then, after being kept for 2 min in such temperature, the samples were cooled to −50 °C and reheated to 120 °C after a 2-min isotherm. The heating and cooling rate was 10 °C/min.

## 3. Results and Discussion

### 3.1. Transport across PIMs Doped with Acac

To determine the optimum pH of the feed phase and composition of the receiving phase, a preliminary transport test with PIMs doped with 20% of ACAC (**2**) was performed.

The pH of the feed phase was varied by addition of an ammonium buffer. The zinc ions concentration in the feed phase was measured after 24 h of the transport process. The zinc amount transported across the membrane was calculated from the formula:

(1) $$RF=\frac{c}{c_{0}}\cdot 100\%$$

where:

- *RF*–recovery factor of zinc ions;
- *c_0_*–initial concentration of metal ions in the feed phase, mol/L;
- *c*–concentration of metal ions in the receiving phase after time t, mol/L.

It was found that the optimum pH of the feed phase for effective recovery of zinc (96%) was 9.62.

An excess of ammonia dissolves zinc hydroxide, which is formed in high pH, leading to the formation of ammine complexes:

Zn^2+^ + 2 OH^−^ → Zn(OH)_2_ (2)

Zn(OH)_2_ + 4 NH_3_ → [Zn(NH_3_)_4_]^2+^ + 2 OH^−.^ (3)

At higher pH (of at least 9.6), the ACAC molecules which are present in the membrane become dissociated:

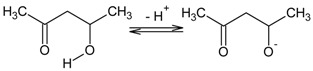
(4)and are able to form stable chelate complexes with Zn(II) ions. Such pH enables the metal ions to be transported across the PIMs.

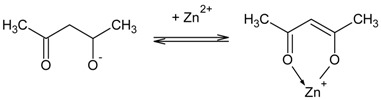
(5)

The amount of the recovered zinc also depends on the composition of the receiving phase. The effect of receiving phase composition on transport through the PIMs was evaluated using an ammonium buffered solution of pH = 9.65, distilled water (pH = 6.94) and 0.5 M nitric acid (pH = 1.34). It was found that after a 24-h process of Zn(II) ion transport across PIMs doped with 20% ACAC, the obtained values of Zn recovery were 0.11%, 23.54%, and 4.14%, respectively.

In the subsequent Zn(II) ion (5mM) transport experiments, involving PIMs doped with acac, a pH value of the feed phase was 9.62 (NH_4_NO_3_:NH_3_) and the receiving phase was a distilled water at pH 6.94.

The results obtained after 24 h of Zn(II) transport across PIMs doped with acac in relation to various amounts of carrier are shown in Table 2.

The highest recovery of zinc(II) (99.65%) was obtained in the case of membrane 2, containing 20% of ACAC.

### 3.2. Transport across PIMs Doped with D_2_EHPA

D_2_EHPA is an acidic extractant. During the transport of Zn(II) ions across membranes doped with D_2_EHPA, extraction and re-extraction take place at the same time, as shown by the reaction equations:

- at interface: feed phase/membrane:
  Zn(II) + 3/2(HR)_2_ = ZnR_2_·HR + 2H^+^ (6)
- at interface: membrane/receiving phase:
  ZnR_2_·HR + 2H^+^ = Zn(II) + 3/2(HR)_2_ (7)

where HR denotes carrier (D_2_EHPA) molecule.

The compositions of the feed phase and the receiving phase for PIMs doped with D_2_EHPA were the same as those reported by Kolev [37]. The feed phase contained 5 mM Zn(II) and 4 mM NaNO_3_, its pH was maintained at 1.61 using HNO_3_, the receiving phase was 0.1 M HNO_3_.

The results of the experiments after 24 h of Zn(II) transport across PIMs doped with D_2_EHPA for various carriers contents are shown in Table 3.

The data in the Table 3 indicate that zinc(II) recovery increases with increasing concentrations of the D_2_EHPA carrier in the membrane. The highest values of RF (56.33%) were obtained for the membrane composed of 35% PVC, 5% DAO, and 60% D_2_EHPA.

### 3.3. Comparision of Acac and D_2_EHPA in the Transport across PIMs

The transport of ions by means of carriers can be divided into following stages:

- formation in the feed phase of a complex composed of the carried substance and the carrier;
- diffusion of the complex across the membrane into the receiving phase;
- decomposition of the complex compound and release of the transported substance into the receiving phase [9].

It was found that the maximum recovered amount of zinc(II), which depends on the carrier in the membrane, for PIMs doped with acac and with D_2_EHPA was 99.6% and 56.33%, respectively. 

The maximum recovery of zinc(II) requires a 60% content of D_2_EHPA in the membrane while in the case of membranes with acac, a 20% carrier content is sufficient in order to obtain the same effect.

An effective transport of zinc ions takes place in acidic conditions for D_2_EHPA and in alkaline conditions for acac. The membranes can be used for zinc recovery from solutions obtained after leaching metal-bearing waste by means of an acid or ammonia. The choice between these two types of membranes for zinc recovery depends on the pH of the solution from which the ions are to be extracted. Zinc recovery by means of PIMs doped with 20% acac in alkaline conditions is twice as high as in the case of PIMs doped with 60% D_2_EHPA in acidic conditions.

### 3.4. Membrane Morphology

Membrane microstructure is one of major aspects affecting the separation of metal ions [42,43]. Supported liquid membranes (SLM) have a specific structure, depending on the particular type polymer film (polypropylene, polyamide, or other ones). Commercially available membrane sheets (such as Celgard 2400, Accurel, Goretex) have strictly defined parameters, namely: thickness, porosity, and pore size. In the case of PIMs, which are prepared by pouring out a polymer solution containing a plasticizer and carrier, the resulting membrane may have a different structure [44,45,46,47], depending on the type and concentration of the reactants [48]. Among all the techniques available for the examination of the surface of PIMs, scanning electron microscopy (SEM) and atomic force microscopy (AFM) are the most useful.

#### 3.4.1. PIMs Characterization by AFM 

Crucial information on the membrane uniformity, roughness, and pore size was obtained by means of AFM. The AFM topographic images of statistically selected membrane areas of all membranes were recorded. 

**Figure 2:**
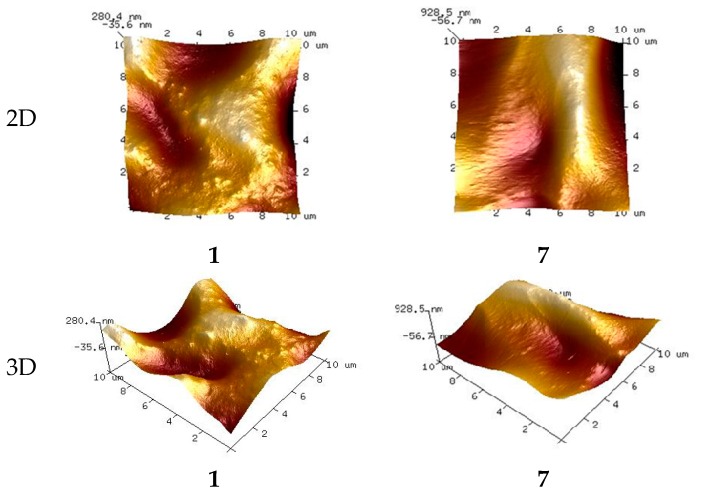
2D and 3D-atomic force microscopy (AFM) images of PIMs with no carriers: (**1**) and (**7**).

**Figure 3:**
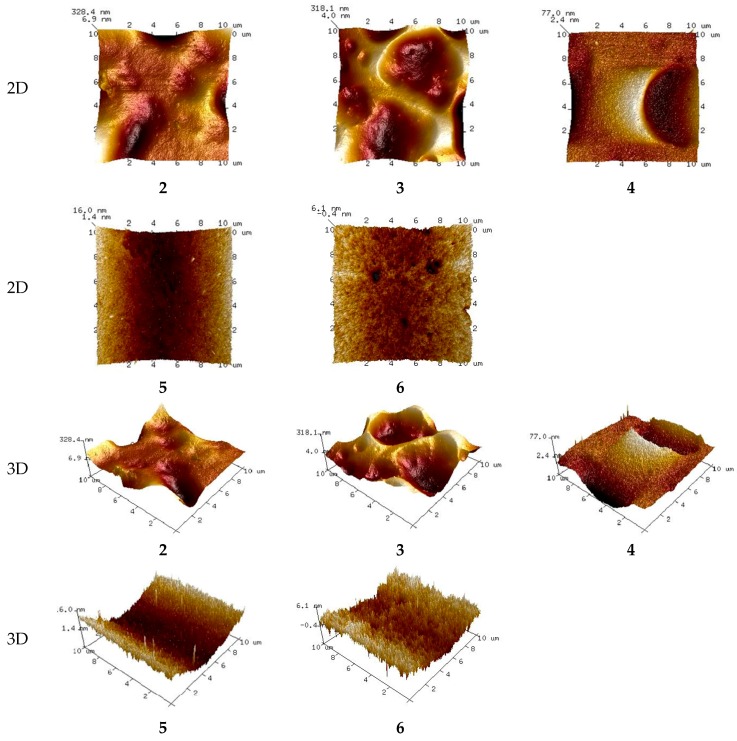
2D and 3D-atomic force microscopy (AFM) images of PIMs with an increasing percentage of carrier (acac): (**2**), (**3**), (**4**), (**5**), and (**6**), the composition of membranes is given in Table 2.

**Figure 4:**
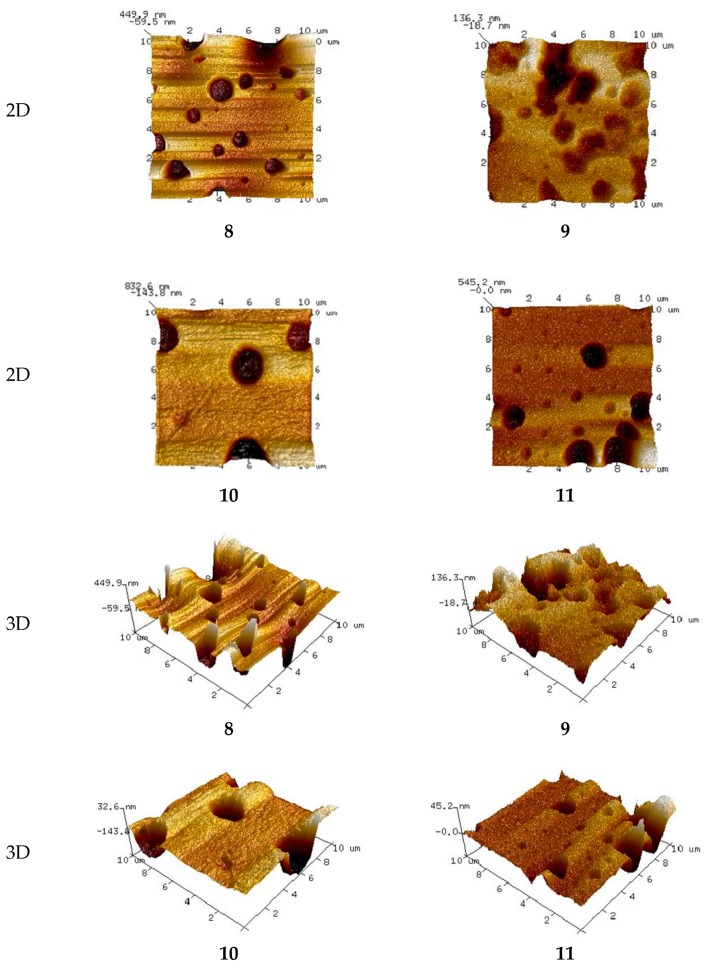
2D and 3D-atomic force microscopy (AFM) images of PIMs with an increasing percentage of carrier (D_2_EHPA): (**8**), (**9**), (**10**), and (**11**), the composition of membranes is given in Table 3.

The 2D and 3D images of membranes (**1** and **7**) with no carrier, with acac and with D_2_EHPA as carrier are shown in Figure 2, Figure 3 and Figure 4, respectively.

The membranes with no carrier are smooth, without visible pores (Figure 2).

The AFM images in Figure 2, Figure 3 and Figure 4 indicate that the carrier distribution in the investigated membranes, after the evaporation of solvent, is homogeneous throughout the entire surface. The surface morphology of the membrane is characterized by rough areas. This is due to either a different speed of the solvent evaporation or the membrane having a porous structure, where the pores were filled DAO, ACAC or D_2_EHPA. The uniform parts of the membranes have well-defined pores. The pores are clearly visible as small, dark areas.

#### 3.4.2. Membrane Pore Size

The pore diameters and roughness of the PIMs were calculated using the Nanoscope Analysis 1.40 Software (Billerica, USA). In both membrane types the number of pores is rather small. The pore size in the membranes with no carrier (**1** and **7**) ranges between 3.403 and 7.215 μm and between 4.831 and 7.445 μm, respectively. The membranes have different concentrations of the DAO plasticizer. As expected, the pore sizes in the membrane with 5% DAO are greater than those in the membrane with 10% DAO. The introduction of carrier into the membranes affected pore dimensions. For the membranes with acac as carrier, the pore sizes increased with increasing ACAC content in the membrane, from 3.201–5.133 μm in the case of the membrane with 20% ACAC (**2**) to 6.400–6.628 μm in the case of the PIM with 70% acac (**6**). In the membranes with D_2_EHPA as carrier, the pore sizes decreased negligibly with increasing D_2_EHPA content in the membrane, from 4.831–7.445 μm for the membranes with 20% D_2_EHPA (**8**) to 2.638–2.839 μm for the PIM with 60% D_2_EHPA (**11**).

The most effective zinc transport was observed in relation to membranes **2** (RF = 99.65%) and **11** (RF = 56.33%), in the case of which the pore sizes were the smallest among the investigated membranes. The membranes with more than 20% ACAC content (**3**–**6**), during the transport of Zn(II) ions suffered from fouling of surfaces by substances depositing in the pores (fouling phenomenon). This resulted in higher membrane resistance, leading to lower efficiency of the transport process [6,49,50].

#### 3.4.3. Membrane Roughness

Mean roughness values for both membrane types, calculated based on three independent measurements, are shown in Table 4. 

Carriers such as acac and D_2_EHPA tend to crystallize in the membrane, causing roughness and diversified porosity. In addition, D_2_EHPA may function as a plasticizer and its molecules preferentially migrate to the membrane surface, increasing its roughness. An increase in roughness due to the presence of carrier, which could act as a plasticizer, was observed in relation to CTA-based membranes with the commercial carrier TOA [51] as well as in the case of PVC-based membranes with Aliquat 336 as carrier [42]. This was also reported in the works [41] concerning the application of such membranes in surface water treatment. 

Although the two polymer membrane types (with ACAC and D_2_EHPA as a carriers) have different roughness, the values are very low, in the range of a few nanometers. Surface roughness accounts for local flowrate problems, therefore, the membrane surface should be as uniform and smooth as possible.

The determined roughness values reflect the pore numbers in the test membranes and correspond well with the results of zinc(II) ion transport.

### 3.5. Characterization of Mechanical Properties of PIMs

#### 3.5.1. Membrane Thickness

Mean thickness of the membranes and standard deviations are shown in Table 5.

As shown in Table 5, the membranes with acac as carrier (**2**–**6**) were thicker than those with D_2_EHPA as carrier (**8**–**11**). In the case of both membrane types, thickness was observed to decrease with increasing carrier percentages.

#### 3.5.2. Resistance to Static Extension

The results of the tests of mechanical properties for selected membranes are shown in Table 6.

The highest modulus of elasticity at static extension was determined for the PVC-based membrane with 5% DAO with no carrier (**7**) and the PVC-based membrane with 5% DAO and 20% D_2_EHPA (**8**). These moduli equaled 794 and 747 MPa, respectively, for **7** and **8**. The other membranes were characterized by clearly lower values of Young’s modulus. Its value was the lowest in relation to the membranes containing acac as carrier, for instance membrane **2** (36 MPa), and membrane **1** with 10% DAO with no carrier (61 MPa). The membranes which were characterized by lower stiffness clearly showed higher values of strain at break, namely 240% and more, while for the membranes with higher value of tensile modulus the values of strain at break were lower than 200%.

**Figure 5:**
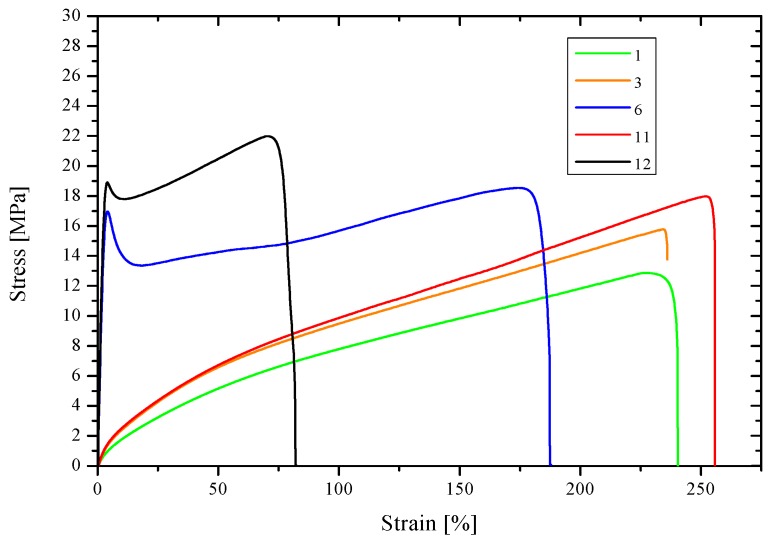
Examples of extension curves for the test membranes. The composition of membranes is given in Table 2 and Table 3.

Moreover, membranes **7** and **8** have a different stress-strain curve profile, with a clearly visible yield point (Figure 5). In the other cases, no maximum value was observed on the curves showing the stress-strain relationship.

The test samples of membrane **8** were observed to have the optimum tensile strength (18.1 MPa).

Tensile strength, however, is not directly linked with the value of Young’s modulus, as proved in the case of membrane **1**: its tensile strength is 17.5 MPa even though its rigidity is quite low.

#### 3.5.3. Membrane Glass-Transition Temperature

In relation to the membranes where acac is the carrier (**2**–**6**) and to the one with 10% of plasticizer (**1**) and no carrier, glass-transition temperature was not recorded between −50 and 120 °C. For membranes **8**–**11** with D_2_EHPA as carrier, glass-transition temperature was in the range between 62.9 and 71.2 °C and increased with an increase in the carrier content in the membrane.

## 4. Conclusions

PIMs based on PVC with no carrier form a thin film with no visible pores. The membranes have a porous structure only after an addition of ACAC or D_2_EHPA. The pores are irregular and few micrometers in size. 

In the case of membranes with ACAC as carrier, pore sizes increase with increasing content of ACAC in the membrane. In contrast, in the case of membranes with D_2_EHPA as carrier, pore sizes slightly decrease as the D_2_EHPA content in the membrane increases.

The two investigated polymer membrane types are characterized by different, though in both cases very low, values of roughness, amounting only to a few nanometers. Surface roughness accounts for local flowrate problems, therefore, the membrane surfaces should be uniform and smooth as possible.

Zinc ions are best transported through membranes with 20% acac or 60% D_2_EHPA. They have the lowest pore sizes.

The zinc ion transport is effective in acidic conditions for D_2_EHPA and in alkaline conditions for ACAC. The membranes can be used for zinc recovery from solutions obtained after leaching metal-bearing waste with an acid or ammonia. The choice between these two types of membranes for zinc recovery depends on the pH of the solution from which the ions are to be extracted. Zinc recovery by means of PIMs doped with 20% ACAC in alkaline conditions is twice as high as in the case of PIMs doped with 60% D_2_EHPA in acidic conditions.

In the case of membranes containing more than 20% ACAC, surface fouling was observed as substances deposited in the pores during the transport of Zn(II) ions. This resulted in higher membrane resistance, leading to lower efficiency of the transport process.

In relation to membranes with D_2_EHPA as carrier, zinc recovery increases with increasing concentration of the carrier in the membrane.

## Figures and Tables

**Table 1:** Compositions of investigated membranes.

Membrane, No.	1	2	3	4	5	6	7	8	9	10	11
Matrix, PVC, %	90	70	50	40	30	20	95	75	55	45	35
Plasticizer, DAO, %	10	10	10	10	10	10	5	5	5	5	5
Carrier	acac						D_2_EHPA				
Carrier, %	0	20	40	50	60	70	0	20	40	50	60

**Table 2:** Recovery factors (RF) for competitive transport of Zn(II) ions across polymer inclusion membranes (PIMs) with acac as a carrier. Membrane: PVC, 10% DAO, and acac. Feed phase: cZn(II) = 5 mM, pH = 9.62; receiving phase: water.

Membrane No.	1	2	3	4	5	6
% ACAC	0%	20%	40%	50%	60%	80%
RF, %	0.51	99.65	23.54	16.64	12.45	6.29

**Table 3:** Recovery factors (RF) for competitive transport of Zn(II) ions across PIMs with D_2_EHPA as a carrier. Membrane: PVC, 5% DAO, and D_2_EHPA. Feed phase: *C*_Zn(II)_ = 5 mM, pH = 1.61; receiving phase: 0.1 M HNO_3._

Membrane No.	7	8	9	10	11
% D_2_EHPA	0%	20%	40%	50%	60%
RF, %	0.95	1.80	25.71	44.27	56.33

**Table 4:** Mean roughness (RF) of PIMs.

Membrane No.	1	2	3	*4*	5	6	7	8	9	10	11
RF, nm	2.91	4.71	3.86	*2.23*	3.55	1.66	1.62	2.42	3.09	5.82	6.15

The composition of membranes is given in Table 2 and Table 3.

**Table 5:** Mean thickness of membrane test samples.

Membrane No.	1	2	3	4	5	6	7	8	9	10	11
Mean membrane thickness, mm	0.242	0.292	0.281	0.273	0.271	0.269	0.185	0.194	0.196	0.187	0.179
Standard deviation	0.050	0.038	0.041	0.046	0.011	0.017	0.080	0.030	0.043	0.018	0.047

The composition of membranes is given in Table 2 and Table 3.

**Table 6:** Mechanical properties of selected membranes, as determined in static extension tests at 20 °C.

Membrane	Young’s Modulus (Mpa)	Tensile Strength (Mpa)	Strain at Break (%)
**1**	61	17.5	240
**2**	36	14.6	279
**4**	58	15.7	264
**7**	794	17.1 *	127
**8**	747	18.1 *	191

* Yield stress. The composition of membranes is given in Table 2 and Table 3.
